# Zebrafish Models of Photoreceptor Dysfunction and Degeneration

**DOI:** 10.3390/biom11010078

**Published:** 2021-01-09

**Authors:** Nicole C. L. Noel, Ian M. MacDonald, W. Ted Allison

**Affiliations:** 1 Department of Medical Genetics, University of Alberta, Edmonton, AB T6G 2H7, Canada; macdonal@ualberta.ca (I.M.M.); ted.allison@ualberta.ca (W.T.A.); 2 Department of Ophthalmology and Visual Sciences, University of Alberta, Edmonton, AB T6G 2R7, Canada; 3 Department of Biological Sciences, University of Alberta, Edmonton, AB T6G 2E9, Canada; 4 Centre for Prions and Protein Folding Diseases, University of Alberta, Edmonton, AB T6G 2M8, Canada

**Keywords:** Danio rerio, inherited photoreceptor disease, retinitis pigmentosa, Leber congenital amaurosis, cone-rod dystrophy, cone dystrophy, choroideremia, macular degeneration, retinal neovascularization, regeneration

## Abstract

Zebrafish are an instrumental system for the generation of photoreceptor degeneration models, which can be utilized to determine underlying causes of photoreceptor dysfunction and death, and for the analysis of potential therapeutic compounds, as well as the characterization of regenerative responses. We review the wealth of information from existing zebrafish models of photoreceptor disease, specifically as they relate to currently accepted taxonomic classes of human rod and cone disease. We also highlight that rich, detailed information can be derived from studying photoreceptor development, structure, and function, including behavioural assessments and in vivo imaging of zebrafish. Zebrafish models are available for a diversity of photoreceptor diseases, including cone dystrophies, which are challenging to recapitulate in nocturnal mammalian systems. Newly discovered models of photoreceptor disease and drusenoid deposit formation may not only provide important insights into pathogenesis of disease, but also potential therapeutic approaches. Zebrafish have already shown their use in providing pre-clinical data prior to testing genetic therapies in clinical trials, such as antisense oligonucleotide therapy for Usher syndrome.

## 1. Introduction

Vision is possible due to specialized light-detecting cells in the eye called photoreceptors. The degeneration of photoreceptors or the retinal pigment epithelium (RPE), essential retinal support cells, results in permanent vision loss and blindness. Photoreceptor diseases are extremely diverse, which creates challenges for development of treatments to prevent or reverse vision loss [1]. The zebrafish model provides opportunity to model diverse blinding diseases and elucidate therapeutic avenues. This review highlights the utility of zebrafish for modelling inherited photoreceptor disease by: (i) introducing photoreceptors and photoreceptor conditions with a genetic component, (ii) outlining zebrafish photoreceptor organization and tools to assess photoreceptor health, (iii) summarizing zebrafish models of photoreceptor disease, (iv) discussing how these models are informative for human disease, and (v) presenting the therapeutic contributions of these models. Ultimately, zebrafish provide a valuable platform for defining disease mechanisms and testing therapeutics that can be used in the clinic.

## 2. Photoreceptors and Their Diseases

**Figure 1:**
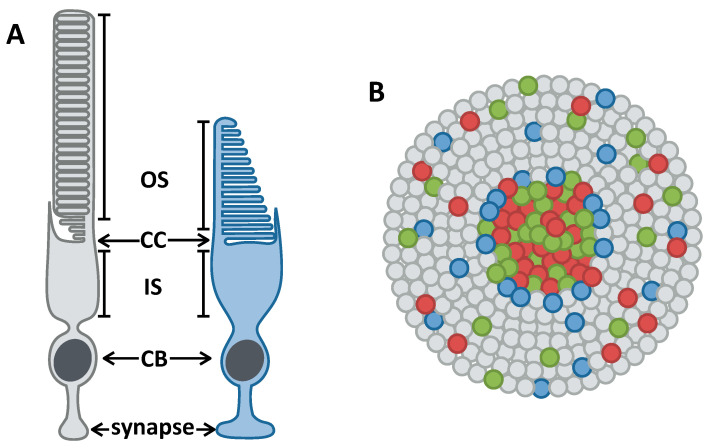
Anatomy of rod and cone photoreceptors and their organization in the human retina. (**A)** Cartoons of a rod (grey) and cone (blue) photoreceptor. Photoreceptors have an outer segment (OS) that is packed with light-sensitive opsin proteins, a connecting cilium (CC) that connects the OS with the mitochondria-rich inner segment (IS), a cell body (CB), and a synapse. (**B**) Cartoon of the human photoreceptor mosaic. Humans have three types of cones: red, green, and blue, depicted in those respective colours. The peripheral retina is rod-dense with cones interspersed throughout, while the central retina is cone-dense.

Photoreceptors are sensory neurons responsible for converting light into signals that are transmitted to the brain for interpretation, allowing the perception of visual information. There are two types of retinal photoreceptors, named after their distinct morphologies: cones, which are maximally sensitive to particular wavelengths of light; and rods, which are extremely sensitive to low levels of light and can detect single photons. Cones allow for high-acuity daytime and colour vision, while rods facilitate vision under dim-light and night conditions. Rods and cones have an outer segment (OS), the light-sensitive part of the photoreceptor; an inner segment (IS), a specialized compartment packed with mitochondria that is connected to the OS by a structure called the connecting cilium (CC); a cell body; and a synaptic terminal, where downstream cells innervate the photoreceptors (Figure 1A). The OS is a modified cilium that is responsible for the photoreceptor names—cones have a cone-shaped OS, while rods have a rod-shaped OS. The OS contains many membranous discs that are packed with opsin, the light-sensitive protein that triggers a signalling cascade, which results in signal transmission to downstream neurons. In cones, OS discs are contiguous with the plasma membrane, whereas rod OS discs are internalized within the plasma membrane.

The human retina has three cone photoreceptor subtypes: blue light-sensitive (*OPN1SW1*), green light-sensitive (*OPN1MW1*), and red light-sensitive (*OPN1LW1*) cones [2,3]. The photoreceptors are organized such that the peripheral retina is rod-dense with cones interspersed throughout, and the central retina is cone-dense (Figure 1B) [4,5]. This cone-dense central region is called the macula, in the middle of which is a pit that contains exclusively cone photoreceptors, known as the fovea [6]. The fovea is important for high-acuity central vision. Fovea-like structures are rarely seen outside of primate species.

Photoreceptors are supported by the RPE. The RPE plays many essential roles in the retina, including maintenance of the blood–retina barrier, absorption of stray photons, transport of materials to and from the underlying choroid vessels, phagocytosis of shed photoreceptor OS fragments, and recycling of chromophores necessary for the visual cycle [7]. The RPE also secretes factors and signalling molecules important for photoreceptor function and neuroprotection, including pigment epithelium-derived factor (PEDF), which aids in photoreceptor survival [8,9,10].

Photoreceptor dysfunction and degeneration can be caused by mutations in many aspects of the photoreceptor cells, including structural factors, cilium components, phototransduction machinery, and ion channels [1]. In addition, RPE dysfunction leads to photoreceptor disease as the RPE has essential roles in photoreceptor maintenance and function. The complexities of photoreceptor development, function, and maintenances can result in extreme genetic and phenotypic heterogeneity for photoreceptor conditions. Moreover, photoreceptor disease can manifest as part of multi-system conditions, such as ciliopathies [11].

In the following sections, photoreceptor diseases with a genetic component are described. Inherited photoreceptor diseases cumulatively affect one in 2000 to one in 3000 individuals, and age-related macular degeneration, a multifactorial condition with a genetic component, is the leading cause of vision loss in the ageing population [12,13,14]. Vision loss has an immense impact on quality of life, and unfortunately these conditions have few effective therapies. This necessitates animal models of photoreceptor disease, as targeted therapeutics cannot be developed without an understanding of disease pathology and underlying mechanisms.

### 2.1. Retinitis Pigmentosa

Retinitis pigmentosa (RP) is a group of rod degenerative diseases, characterized by night blindness and peripheral vision loss. RP is the most common inherited photoreceptor condition, affecting one in 3500 to one in 5000 individuals. During RP progression, the RPE becomes degenerate and pigment granules can translocate into the retina, resulting in a feature called bone spicule. Cones are spared initially in RP, but eventually degenerate, leading to total blindness. Over 70 genes have been associated with RP, and RP can be inherited in an autosomal recessive, dominant, X-linked, digenic, or mitochondrial manner [1]. Additionally, RP can be caused by mutations that directly affect the rod photoreceptors or the RPE. RP can manifest alone or in combination with extraocular phenotypes in syndromes.

### 2.2. Leber Congenital Amaurosis

Leber congenital amaurosis (LCA) is a severe, early-onset disease of the RPE and photoreceptors. LCA is caused by mutations in essential RPE and photoreceptor genes. The genes most commonly mutated in LCA patients are *RPE65*, *GUCY2D*, and *CEP290* [15,16,17,18]. The *RPE65* gene produces an RPE-specific retinoid isomerohydrolase, which is essential for recycling of components required for the photortransduction cascade [19]. *GUCY2D* produces the protein retinal guanylate cyclase-1 (RETGC1), which is also needed for phototransduction [20,21]. *CEP290* encodes a centrosomal protein important for development of the centrosome and cilium [22].

### 2.3. Choroideremia

Choroideremia is an X-linked condition characterized by degeneration of the photoreceptors, RPE, and underlying choroid vasculature. Choroideremia is caused predominantly by mutations in the gene *CHM*, which encodes the protein REP1 [23]. REP1 posttranslationally modifies Rab proteins, which are essential for Rab protein function. Defects in Rab protein function would impact processes in many retinal cell types, but there is evidence from animal models that the RPE defects lead to photoreceptor degeneration when REP1 is lost [24,25].

### 2.4. Cone-Rod Dystrophy

Cone-rod dystrophies (CORD) are conditions where both the cones and rods are affected. CORD patients can present with visual acuity loss, light sensitivity, colour vision defects, central vision loss, and partial peripheral vision loss.

### 2.5. Enhanced S-cone Syndrome

Enhanced S-cone syndrome is a unique condition, as it presents with increased photoreceptor function. Enhanced S-cone syndrome is caused by mutations in the transcription factors *NR2E3* or *NRL*, which are required for rod development [26,27,28,29]. Patients with enhanced S-cone syndrome therefore have cone-only retinas with enhanced blue cone sensitivity and experience night blindness and photoreceptor degeneration [26].

### 2.6. Cone Dystrophy

Cone dystrophies are conditions that affect the cone photoreceptors, leading to their dysfunction and degeneration. Cone dystrophies typically present with visual acuity defects, colour vision abnormalities, and reduced cone responses, measured by electroretinography (ERG). Although extremely rare, there are reported cases of peripheral cone dystrophies, whereby the cones in the peripheral retina are more affected than those in the central macula [30].

Macular degenerations involve loss of cones in the macula and leads to visual acuity decline, colour vision disturbances, and central vision loss. Cone loss can result in macular atrophy, which is visible on routine fundoscopy imaging. There are many different types of macular degeneration, the most common of which is age-related macular degeneration (AMD).

AMD is the leading cause of vision loss in the ageing population [12]. AMD is a multifactorial condition, and a combination of genetic and environmental factors influence a person’s likelihood of developing AMD. There are two forms of AMD: dry and wet. Dry AMD is the most common, characterized by geographic atrophy that results from the degeneration of RPE, photoreceptors, and choroid vessels. Dry AMD can progress to wet AMD, which is characterized by choroidal neovascularization. The aberrant vessels are fragile and prone to leaking or bursting, which can lead to fluid buildup and retinal scarring. Lipid deposits, called drusen and subretinal drusenoid deposits, are hallmark features of AMD. Drusen develop between the RPE and underlying choroid vasculature, while subretinal drusenoid deposits occur between the photoreceptor OSs and RPE. Although characteristic features of AMD, the impact that drusen and subretinal drusenoid deposits have on the retina and photoreceptor biology is unclear.

### 2.7. Achromatopsia

Achromatopsia is a condition characterized by lack of colour vision. Achromatopsia can either be complete or incomplete; individuals with complete achromatopisa have no functioning cone photoreceptors, while those with incomplete achromatopsia have partial cone function. Achromatopsia is generally caused by mutations in cone-specific phototransduction machinery or ion channels that prevent the cones from relaying messages to downstream neurons [1,31].

## 3. Zebrafish as a Model for Photoreceptor Disease: Photoreceptor Features, Function and Morphological Assessment, and Model Generation

Zebrafish are a powerful model for photoreceptor development and disease. The zebrafish retina is remarkably conserved to the mammalian retina in terms of development, structure, and function. Researchers can study cone photoreceptor development and disease in zebrafish, which is challenging in nocturnal rodent models. There are many tools for assessing zebrafish visual responses, photoreceptor function, and morphology in live animals. Additionally, there are well-established technologies for generating transgenic and mutant animals. These features are outlined below. For reviews that compare retinal features of different animal species used to model disease, see [32,33].

### 3.1. The Zebrafish Visual System

Zebrafish have a visual system that develops rapidly and larval zebrafish can be assessed for visual function at early ages. Zebrafish embryos develop externally and embryos are transparent, meaning that visual system development can be easily observed. Eye field patterning begins at 28 h post fertilization (hpf), and the developing retina expresses opsin genes as early as 51 hpf [34,35]. At 5 days post fertilization (dpf), zebrafish can carry out complex visually-mediated behaviours such as prey capture [36], meaning that larvae can be assessed for visual defects very early in life.

The zebrafish retina is cone-rich, similar to the human macula. This is a huge benefit since other commonly used models, such as mice and rats, are nocturnal with few cones and no macula-like region. The abundance of cone photoreceptors allows for modeling of cone diseases in zebrafish. However, there are nevertheless notable differences between zebrafish and humans. Zebrafish have double cones, which are not observed in the mammalian retina. Additionally, the zebrafish photoreceptor mosaic is organized differently than the human mosaic, and while it can model general cone dystrophies, cannot accurately recapitulate macular degeneration. Although zebrafish have uneven distributions of cones at some stages, their retina does not have a macular region with increased cones and a central cone-only foveal pit [37]. It is important to note that although zebrafish do not have a retinal region that is structurally similar to the fovea, larvae do have a region that is important for high-acuity vision and is therefore functionally similar to the human fovea [38].

**Figure 2:**
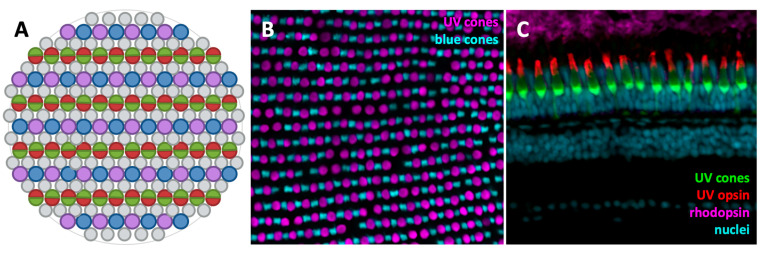
Zebrafish photoreceptor organization. (**A**) Cartoon depiction of the adult zebrafish photoreceptor mosaic. UV and blue cones, depicted in purple and blue respectively, alternate in their rows while red and green double cones alternate in neighbouring rows. Rods are studded throughout. (**B**) Fluorescent image of a flat-mounted adult transgenic zebrafish retina, with GFP expressed in UV cones (magenta) and mCherry expressed in blue cones (cyan). The alternation of UV and blue cones in their rows is apparent. (**C**) Immunofluorescent image of a cryosectioned adult Tg(*sws1:GFP*) zebrafish retina with GFP in UV cones (green). UV opsin (red) and rhodopsin (magenta) are labelled, as well as nuclei (cyan).

The zebrafish photoreceptor mosaic is differentially organized between larval and adult stages. Zebrafish have four cone subtypes: ultraviolet (*sws1*, homolog of mammalian *OPN1SW*), blue (*sws2*), green (*rh2*), and red (*lws*, homolog of mammalian *OPN1MW* and *OPN1LW*). The green and red cones exist as physically fused double cones. Larval zebrafish have a cone-dominant retina with few rods [37,39]. As the larval zebrafish age, they develop more rods, and in adulthood there are roughly equal proportions of cones and rods [37]. Adult zebrafish photoreceptors are organized into a highly structured a row mosaic, with UV and blue cones alternating in their rows, and red and green cones alternating in neighbouring rows [37,40] (Figure 2A,B). Some opsins have multiple copies that are differentially expressed according to retinal location and age. For example, zebrafish have four green opsin genes (*rh2-1*, *rh2-2*, *rh2-3,* and *rh2-4*), two red opsin genes (*lws1* and *lws2*), and two rhodopsin genes (*rho* and *rhol*), which have different spatial localizations depending on developmental stage [41,42,43]. There are many tools available for visualizing zebrafish photoreceptors, including transgenic lines expressing fluorescent proteins in specific cell types and antibodies for immunolabelling (Figure 2B,C). A subset of commonly used transgenic lines (Table 1) and antibodies (Table 2) are highlighted here; available zebrafish tools are catalogued on the Zebrafish Information Network (ZFIN) [44]. The well-characterized promoters in these transgenes can also be utilized to drive expression of other genes, such as disease or cell death genes.

**Table 1:** Examples of common zebrafish transgenic lines with labelled photoreceptors.

Transgene	Description	Reference
Tg(*3.2gnat2:eGFP*)	Enhanced GFP expressed in all cone photoreceptors	[45]
Tg(*rho:eGFP*)	Enhanced GFP expressed in rod photoreceptors	[46]
Tg(*sws1:GFP*)	GFP expressed in UV cones	[47]
Tg(*sws2:mCherry*)	mCherry expressed in blue cones	[48]

**Table 2:** Examples of commonly used antibodies that label zebrafish photoreceptors.

Antibody	Antigen	Labels	Reference
4c12	N/A	Rods	[49]
10c9.1	UV opsin	UV cone OSs	[50]
1D4	Red opsin	Red cone OSs	[51]
zpr1	Arrestin3a	Red/green double cones	[52,53]
zpr3	N/A	Rod and double cone OSs	[54]

### 3.2. Visually-Mediated Responses as Diagnostics in Zebrafish

Visual function can be investigated in zebrafish larvae by several behavioural tests. Visually-mediated responses in zebrafish larvae include the optokinetic response (OKR), optomotor response (OMR), and visual motor response (VMR). OKR tracks the eye movements of the larvae in response to visual stimuli. OKR involves immobilizing a larvae and placing the animal inside of a drum with moving black and white stripes [55,56,57,58]. As the drum moves, the zebrafish’s eyes will track the movement then saccade back to the origin [55,56,57,58]. OKR can be detected as early as 3 dpf [57,58]. OMR uses a visual stimulus to elicit a swim response. Larvae are placed inside narrow tracks on top of a flat screen [58,59,60], a stimulus of moving lines plays on the screen, and the fish swim in the direction of the moving stimulus [58,59]. OMR is detectable as early as 4 dpf, but is more robust at 6–7 dpf [59]. VMR is a visually-mediated startle response triggered by rapid changes in light. Individual larvae are placed into wells of a 96-well plate and then the plate is inserted into a recording chamber with lights that can be turned on and off [61]. Larvae will become active when the lights come on, followed by returned to baseline; similarly, when the lights are turned off, there is an increase in activity followed by a return to baseline. VMR can be elicited from larvae as early as 4 dpf.

### 3.3. Retinal Imaging and Functional Assessement for Zebrafish

Zebrafish have many tools available for the assessment of photoreceptor health in live animals. These techniques include optical coherence tomography (OCT), ERG, calcium imaging, and fundoscopy.

OCT is a live imaging technique that uses the differential reflectivity of the various retinal cell types to capture images of the retinal layers and photoreceptor mosaic. Photoreceptor ISs are highly reflective due to the abundance of densely packed mitochondria, thereby facilitating visualization of the photoreceptor layer [62]. Refractive properties of the zebrafish lens cause zebrafish OCT to be very high resolution, and individual photoreceptor ISs can be observed [63,64]. En face projections provide a view of the photoreceptor mosaic.

Electroretinography is an electrophysiological method for assessing retinal function. ERG involves placing an electrode on the subject and using light stimuli to elicit a response. The testing conditions and light stimuli can be modified to assess specific photoreceptor types. ERG has been utilized to assess both larval and adult zebrafish [65,66,67,68,69]. Calcium imaging techniques can also be used to assess photoreceptor activity in transgenic zebrafish larvae expressing a Ca^2+^ sensor fused to a fluorescent protein [70,71]. When Ca^2+^ is present in the cell, such as during neuronal activity, the protein undergoes a conformational change that increases fluorescence [72,73]. This is a useful tool to assess whether photoreceptor cells are responding to stimuli, although it cannot easily be used in live adult zebrafish due to eye size and significant pigmentation.

Fundoscopy involves shining a light into the eye to observe the back of the eye, termed the fundus. Zebrafish fundoscopy enables visualization of adult zebrafish retinal cells in transgenic animals expressing fluorescent proteins [48]. This can be used to observe progressive photoreceptor degeneration and regeneration.

### 3.4. Generation of Transgenics and Mutants

External fertilization and large clutch size mean that zebrafish embryos are very accessible. This makes generation of transgenic or mutant animals straightforward, as constructs can be injected directly into the single-cell embryo [74]. The generation of transgenic zebrafish can be accomplished using transposase and Tol2 transposable element sites or I-SceI meganuclease [75,76,77]. However, some transgene sequences are susceptible to silencing in zebrafish. The Gal4/UAS transcriptional activation system is a gene/enhancer trap system; the zebrafish-adapted version is KalTA4/UAS [78]. This involves the tissue-specific activation of the KalTA4 transcription factor, which then recognizes the UAS promoter and drives expression of the downstream transgene—for example, a fluorescent protein. Unfortunately, the UAS promoter becomes silenced throughout subsequent generations, and silencing can even be observed in individual fish; larvae may have high levels of expression, but expression can decrease drastically in adulthood due to silencing [79,80]. However, silencing seems to be a relatively uncommon occurrence when transgenes are generated using endogenous zebrafish promoter sequences. Promoter silencing has also created challenges for generation of conditional knockout lines in zebrafish. Cre-lox recombination utilizes Cre recombinase and lox target sites to create conditional loss-of-function alleles. Cre can be expressed ubiquitously or under tissue-specific promoters. Cre-lox has been successfully utilized in zebrafish and there are increasing numbers of tools and zebrafish Cre-driver lines [81,82,83,84,85,86]. However, it has been difficult to find tissue-specific promoters that are not silenced over time, as well as issues activating inducible Cre in zebrafish. In addition, it is challenging to insert lox sites around target genes. As a result, conditional knockout zebrafish are difficult to generate and rare.

There are many means by which mutations have been introduced to zebrafish, including in forward genetic screens using mutagens such as ethylnitrosourea, or targeted gene mutations with zinc finger nucleases (ZFNs), transcription activator-like effector nucleases (TALENs), and clustered regularly interspaced short palindromic repeats (CRISPR)/Cas [87,88,89,90]. However, mutants isolated from forward genetics screens require extensive mapping in order to locate the affected gene(s), and some lines never had a mutated gene identified. ZFN and TALEN editing strategies require fusing a nuclease to a sequence-specific DNA binding domain to target genomic regions; this process can therefore be strenuous, but resulting mutants are unlikely to have off-target mutations introduced. CRISPR/Cas systems are easily adaptable to different target genes, but off-target cutting is difficult to predict, though there are methodologies that help limit off-target mutagenesis [91,92,93,94]. There are nevertheless challenges associated with the zebrafish genome. The teleost lineage underwent a genome duplication event, meaning that many genes have two copies. This creates complexities when making mutants, and functional redundancy between the paralogs can necessitate generation of double mutants. Conversely, there can be sub-specialization of the paralogs. It is not uncommon for one gene to be specific to cones while the other is either specific to rods, have pan-photoreceptor expression, or be expressed elsewhere in the retina or nervous system entirely [95,96,97,98].

### 3.5. Regenerative Response

The mature human retina has very limited capacity to replace lost neurons, meaning that loss of any retinal neurons is irreversible and can lead to vision impairment. Conversely, zebrafish retinas have a robust regenerative capacity [99]. This is both a benefit and challenge, as it permits investigation into mechanisms of neuronal regeneration, but also means that the zebrafish retina responds differently to acute retinal lesions than the human system. Assessment of disease progression can be complicated by regeneration of lost photoreceptors; there may be cases where degeneration is underestimated due to regenerated photoreceptors restoring visual function. It is important for researchers to be aware of how regenerative responses may affect their assessment of retinal damage longitudinally. However, factors necessary for regenerative responses can be pharmacologically blocked or knocked down to allow for disease progression more comparable to humans [100,101,102], although blocking regeneration may cause additional issues with retinal biology. Potential regenerative responses are important to be aware of when investigating models for potential degeneration. Mechanisms underlying the zebrafish retinal regenerative response will be discussed further in Section 5.3.

## 4. Zebrafish Models of Photoreceptor Disease

There are many zebrafish models of photoreceptor disease, including models where degeneration is induced by exposure to light or toxins, transgenic lines expressing deleterious components, and mutants, which are reviewed here.

### 4.1. Light Ablation

Light ablation allows for photoreceptor ablation while the remaining neural retina stays relatively intact [99]. There are also differences in susceptibility of photoreceptor types depending on retina location, potentially due to the amount of light that reaches specific areas [103,104,105,106,107]. Light ablation typically causes generalized destruction of the photoreceptors, though targeted laser ablation can be performed to target specific photoreceptor types [108]. A robust regenerative response occurs post light ablation [109].

### 4.2. Toxic Lesions

#### 4.2.1. Ouabain

Retinal lesion can be achieved with injection of ouabain, which inhibits Na^+^/K^+^-ATPase and thereby reduces available ATP for cellular processes [110]. Dosage determines the retinal layers that are affected. High doses can penetrate far into the retina, damaging many retinal cell types including photoreceptors, while lower doses only reach inner retinal layers [110,111,112]. This method cannot easily be limited to only one cell type, although ouabain could be injected subretinally to cause more targeted photoreceptor and RPE damage. In zebrafish, ouabain has been used successfully to ablate retinal neurons, including photoreceptors, and the resultant damage stimulates a regenerative response [111].

#### 4.2.2. *N*-Methyl-*N*-nitrosourea

*N*-methyl-*N*-nitrosourea (MNU) is an alkylating agent that transfers its methyl group to nucleic acids, resulting in DNA mutations and damage. It is a carcinogen, typically used to induce tumours in cancer models [113,114]. MNU treatment in animals induces photoreceptor cell death, presumably through DNA damage [115,116,117]. MNU treatment primarily affects the rod photoreceptors and therefore produces rod degeneration akin to RP [115]. The acute loss of rods results in a regenerative response in zebrafish [115]. Why MNU treatment is more damaging to rods than cones is unknown.

### 4.3. Hypoxia-Induced Neovascularization

Exposing adult zebrafish to a hypoxic environment results in retinal neovascularization [118]. Angiogenic sprouts being to grow around day 2 and continue to grow throughout hypoxia treatment. Similar to wet AMD neovascularization, the aberrant vessel growth can be minimalized with anti-VEGF treatment. How the neovascularization impacts retina function in adult zebrafish is poorly investigated.

### 4.4. Gene Knockdown (Morpholinos)

Morpholinos are antisense oligonucleotide analogs, usually 20–25 bases in length, which complement and bind mRNA to prevent the production of protein products. Morpholinos can either block mRNA splicing or translation [119,120]. They can be easily introduced to zebrafish embryos by microinjection at the single-cell stage [119]. However, morpholinos can have off-target effects and morpholino experiments need to be carefully controlled. It has been reported that morpholino phenotypes are often not recapitulated in genetic mutants [121]; the reason for this is unclear, but may be due lack of genetic compensation. When there are genomic mutations, there is genetic compensation in response to the genetic lesions. However, this compensation does not occur in a gene knockdown scenario [122]. Despite their limitations, morpholinos provide the opportunity for preliminary assessment of potential gene function when properly controlled, which is extremely valuable for expeditious assessment of putative disease-causing genes.

### 4.5. Transgenic Models

#### 4.5.1. XOPS:mCFP

The Tg(*XOPS:mCFP*) transgenic zebrafish line expresses membrane-targeted cyan fluorescent protein (mCFP) under the *Xenopus* rhodopsin promoter (*XOPS*) [123] (Table 3). The mCFP is overexpressed, resulting in toxicity and rod degeneration. Interestingly, there is no sign of cone death, despite rod loss. This differs from what is observed in humans with RP, where cone degeneration follows rod death. The reason for cone persistence despite rod degeneration in the Tg(*XOPS:mCFP*) zebrafish is unclear, though could be due to the cone-rich environment of the zebrafish retina, compared to the rod-rich human retina. The phenomenon of cone survival in zebrafish provides opportunities to investigate how cone photoreceptors respond to rod loss, and may provide insight into factors contributing to cone death in human RP.

#### 4.5.2. Nitroreductase Ablation

Nitroreductase (NTR, *nfsB* gene) is a bacterial enzyme that can reduce otherwise inert prodrugs, such as metronidazole (MTZ) into DNA cross-linking agents, allowing for the targeted ablation of cells expressing NTR [124]. Importantly, there appears to be no toxic bystander effects as the result of NTR ablation, and neighbouring (non-NTR expressing) cells are not damaged by the treatment [60,124,125,126]. The NTR mechanism of ablation is thus one of the few methods that allows for ablation of targeted cell subtypes. Additionally, as NTR-fluorescent protein fusions have been made, the degeneration of target cells can be followed, and, where possible, regeneration of these cells can also be observed. It is also possible to delay, or prevent, the regeneration of the target cell type by continued treatment with MTZ, since regenerating cells will undergo apoptosis once they begin to express NTR.

The NTR method has been used to ablate UV, blue, and red cones as well as rods in zebrafish [60,125,126,127,128]. Intriguingly, ablation of specific photoreceptor subtypes has revealed thresholds for triggering regenerative responses. In larval zebrafish, NTR-mediated blue cone ablation did not stimulate appreciable amounts of regeneration [127]. However, simultaneously ablating both blue and red cones using the NTR mechanism successfully induced robust regeneration. Conversely, ablating solely UV or red cones successfully stimulated a regenerative response [125,127,129]. Regenerated cones can be of multiple subtypes, and are not restricted to the ablated cone subtype [129]. How NTR ablation of specific cone subtypes triggers regenerative responses in the adult zebrafish retina has not been investigated. The ablation of rods in the adult zebrafish retina demonstrated that there is similarly a threshold of damage that needs to be reached to trigger regenerative responses. Ablating the vast majority of rod photoreceptors stimulated a regenerative response, but incomplete ablation of rods did not [126].

NTR ablation of RPE resulted in RPE degeneration as well as subsequent photoreceptor degeneration [130]. RPE loss by this method triggered a regenerative response, and animals were able to re-establish an RPE monolayer. While regenerative capacity for retinal neurons in zebrafish is well established, this was the first study to show RPE regeneration post degeneration.

#### 4.5.3. Disease Genes

Transgenesis technology allows for insertion of a disease-causing gene from humans or other species into the zebrafish genome. This can be used to generate models of photoreceptor disease, as well as investigate how the mutated protein behaves within and impacts photoreceptor cells.

There are several transgenic zebrafish models of RP-like photoreceptor disease (Table 3). Zebrafish with transgenically introduced mouse rhodopsin with a common RP mutation [131,132,133], Tg(*rho:msRho-P23H-flag*), have notable rod loss by early adulthood [134]. Similarly, a line expressing human rhodopsin with the RP-causing Q344X mutation in rod photoreceptors, Tg(*rho:hsaRHO-Q344X*), has rapid rod degeneration [135,136]. Adcy is an enzyme normally found in the IS that is related to mechanisms of photoreceptor cell death; antagonists of Adcy increase photoreceptor survival in degeneration models [136]. An RP-like transgenic line that expresses Adcy2b with a C-terminal rhodopsin tail, targeting the protein to the OS, undergoes rod degeneration [136]. *PRPF31* is involved in splicing of pre-mRNA and has been associated with RP [137]. Tg(*rho:prpf3-AD5-mCherry*) zebrafish express mutant *prpf31* in rods and have abnormal splicing for a subset of important photoreceptor genes and increased cell death in the photoreceptor layer [138]. A single nucleotide polymorphism in the *HTRA1* promoter region increases an individual’s likelihood of developing AMD [139,140]. Overexpression of HTRA1 in mouse RPE has also been reported to induce choroidal neovascularization, similar to what is observed in AMD [141]. Zebrafish larvae transgenically expressing human *HTRA1* in rod photoreceptors have rod cell death [142]. Of interest, the zebrafish model of RP expressing human mutant rhodopsin have increased *htra1a* expression [142].

There is a transgenic cone-rod dystrophy model with mutant human *GUCY2D*, which produces the protein retinal guanylate cyclase-1 (RETGC1) [143] (Table 3). Cones degenerate in larval zebrafish, followed by rod degeneration. In early adulthood, rods look dysmorphic, but whether rods continue to degenerate in aged adult retina has not been reported.

**Table 3:** Transgenic zebrafish models of photoreceptor disease.

Photoreceptor Disease	Transgene	Photoreceptor Features	Reference
Cone-rod dystrophy	Tg(*3.2gnat2:hsa.GUCY2D-E837D R838S*)	Dysmorphic cones at 5 dpf. 3-month-old animals have a thin photoreceptor layer in the central retina, dysmorphic cones, and less cone and rod staining	[143]
Retinitis pigmentosa	Tg(*rho:hsaRHO-Q344X*)	Rod degeneration detectable at 5 dpf, rapid progression.	[136]
Retinitis pigmentosa	Tg(*rho:msRho-P23H-flag*)	Rod degeneration at 3 months.	[134]
Retinitis pigmentosa	Tg(*rho:prpf3-AD5-mCherry*)	Increased photoreceptor cell death at 5 months.	[138]
Retinitis pigmentosa	Tg(*rho:adcy2b-rho-tail*)	Rod degeneration noted at 14 dpf.	[136]
Retinitis pigmentosa	Tg(*rho:hsaHTRA1*)	Rod degeneration at 5 dpf.	[142]
Retinitis pigmentosa	Tg(*XOPS:mCFP*)	Rapid rod degeneration by 5 dpf. Adults do not have a rod response on ERG.	[123]

### 4.6. Genetic Mutants

Genetic screens and genome editing strategies have fostered the discovery and generation of many zebrafish mutants with photoreceptor phenotypes. Of note, there are instances where the disease phenotype in zebrafish differs from what is observed in human patients with mutations in the same gene. This phenomenon may be due to disparities in the type of genetic lesions present. Loss-of-function mutations are most commonly engineered in animal models, but disease-causing mutations observed in humans may instead be missense, splice site mutations, or compound. Alternatively, these divergences could be related to gene duplication and subspecialization/neospecialization in zebrafish, or differences between the human and zebrafish photoreceptor mosaic.

Mutations in genes affecting cilia or other proteins expressed in many tissue and cell types may have extraretinal phenotypes and early lethality. Only the photoreceptor phenotypes will be focused on here. For previous reviews on zebrafish models of ocular disease, please see [144,145,146,147,148].

Please note that the zebrafish mutants are categorized based on the condition that their phenotype is most similar to, and this may not necessarily align with the phenotypes observed in human patients with mutations in the same gene. In order to be included here, the zebrafish mutant lines had to have an identified mutated gene and present with photoreceptor degeneration, photoreceptor dysfunction, or otherwise fit into a photoreceptor condition category based on retinal findings.

#### 4.6.1. Retinitis Pigmentosa

Mutations in the rod-specific opsin gene, rhodopsin (*RHO*), are a frequent cause of RP [131,132,133,149]. Recently, several *rho* mutant zebrafish lines have been generated which model dominant or recessive RP [150,151] (Table 4; see for list of zebrafish mutant RP models). These animals have rod degeneration beginning soon after rod development and continuing into adulthood [150,151]. Cones are unaffected in *rho* mutants.

RP2 is a cilium-associated protein and its mutation causes X-linked RP [152,153,154,155]. Knockout of *rp2* in zebrafish results in photoreceptor functional defects in larval animals, as well as progressive rod OS degeneration, followed by cone OS degeneration [156]. Mutations in *CERKL*, a gene involved in metabolism of components important for neuron survival, can cause RP [157]. Zebrafish *cerkl* knockout larvae have photoreceptor defects detected by electrophysiological assessment [158]. Young adults have OS defects, primarily affecting the rods, which progress to rod degeneration and eventual cone OS defects. *KIF3B* encodes a kinesin motor involved in transport through the cilium and mutations in *KIF3B* have recently been associated with a ciliopathy that presents with RP [159]. *kif3b* mutant zebrafish have rapid rod degeneration and delayed OS genesis, but cones appear normal [160,161]. RPGRIP1 localizes to the CC and OS and mutations in *RPGRIP1* cause several different photoreceptor diseases, including LCA, RP, and CORD [162,163,164,165]. Zebrafish *rpgrip1* mutants do not have proper rod OS development, and none are observed at 5 dpf [166]. Rod cells were almost entirely absent by 3 months of age. Cones appear normal at 7 dpf, but have functional defects and cone degeneration is clear by 6 months. By 23 months, almost all photoreceptors are lost. Interestingly, double cones degenerate before single cones. *RP1L1* is a photoreceptor cilium-associated protein, mutations in which lead to RP and macular degeneration [167,168,169]. Mutant *rp1l1* zebrafish have rod functional defects at 6 months, which worsen over time [170]. By 12 months, animals have disorganized rod OSs, rod loss, and subretinal drusenoid deposits [170].

*MYO7A* encodes a myosin protein expressed in human photoreceptors and RPE [171]. *MYO7A* mutations lead to a subtype of Usher syndrome, a condition characterized by hearing and vision loss [172,173]. *myo7aa* mutant larvae have decreased photoreceptor function and rod photoreceptor loss [174]. Mutations in *USH2A* also cause a subtype of Usher syndrome [175]. *USH2A* encodes the transmembrane protein usherin, the exact function of which is unknown [175,176]. *ush2a* mutant zebrafish have photoreceptor functional defects in larvae, and progressive photoreceptor degeneration in adulthood [177,178]. Rods begin to degenerate prior to cone degeneration [177].

*her9* is a transcriptional regulator that has increased expression in Tg(*XOPS:mCFP*) retinas undergoing rod degeneration [179]. *her9* mutant larvae have a lower abundance of rods and there is evidence that red cones do not develop appropriately or rapidly degenerate [180]. After rods begin to decrease in number, green cones are reduced in number and appear to have small OSs, while blue and UV cones appear unaffected. Animals do not survive past 13 dpf. *her9* orthologs have not been associated with human photoreceptor disease.

**Table 4:** Zebrafish mutant models of retinitis pigmentosa-like photoreceptor disease.

Gene	Photoreceptor Features	Reference
*cerkl*	Photoreceptor functional defects at 7 dpf. Rod OS defects at 3 months, cone OS defects at 7 months. Notable thinning of the photoreceptor layer and cell death by 12 months.	[158]
*her9*	Decrease in rod photoreceptors at 5 dpf. Few double cones with short OSs at 12 dpf.	[180]
*kif3b*	Delayed OS development. Rapid rod degeneration by 5 dpf.	[160,161]
*myo7aa*	Decreased photoreceptor function at 5dpf. Reduced rods at 8 dpf.	[174]
*rho*	Rod loss observed at 6 dpf. Degeneration continues into adulthood.	[150,151]
*rp1l1*	Rod dysfunction at 6 months. Subretinal drusenoid deposits at 11 months. Photoreceptor loss observed at 12 months.	[170]
*rp2*	Photoreceptor functional defects at 7 dpf. Short rod OSs at 2 months; cone OS defects at 4 months; significant rod OS loss and decreased cone OSs by 7 months.	[156]
*rpgrip1*	No rod OSs at 5 dpf. Cone dysfunction at 7 dpf. Severe rod degeneration by 3 months, followed by cone degeneration. By 23 months, majority of photoreceptors have degenerated.	[166]
*ush2a*	Decreased photoreceptor function at 5–7 dpf and increased photoreceptor apoptosis at 8 dpf. Notable rod OS degeneration at 12 months, cone OS degeneration observed at 20 months.	[177,178]

#### 4.6.2. Leber Congenital Amaurosis

Mutations in genes involved in ciliogenesis initiation, cilia elongation, transport of cilium components, or physiological processes can result in LCA or an LCA-like phenotype in animal models. Intraflagellar transport (IFT) proteins play crucial roles in movement of cargo in the cilium, which can be facilitated by kinesin motors. *ift88* and *ift172* zebrafish mutants have no photoreceptor OS development and the photoreceptors degenerate [181,182,183,184] (see Table 5 for list of zebrafish mutant LCA models). Sensory cilia in general do not develop in these animals [183]. Similarly, *cluap1* (also known as *ift28*) mutant zebrafish have ciliogenesis defects that result in no OS development and rapid photoreceptor degeneration [185]. Fish with mutations in *ift57* develop photoreceptor OSs, although they are shorter than normal, and photoreceptors degenerate soon after developing [184,186]. *ift122* mutant zebrafish have normal photoreceptor development, but similarly undergo degeneration [187]. Zebrafish *kif3a* (kinesin family 3a) mutants fail to develop OSs and photoreceptors rapidly degenerate, and larvae have an extinguished ERG [161,188,189]. *napbb* encodes a protein important for synaptic vesicle fusion [190] and *napbb* mutant photoreceptors undergo cell death almost immediately after specification [191,192]. No photoreceptor layer is detectable at early larval stages, with only a few photoreceptors observed at the ciliary margin [191,192]. *IFT88*, *CLUAP1*, *IFT57*, *IFT122*, *KIF3A*, and *NAPB*, mutations have not been associated with human photoreceptor disease, likely because of essential roles in development. *IFT172* mutations have been associated with RP [193].

*KIAA0586* mutations underlie a ciliopathy called Joubert syndrome, which presents with brain abnormalities and photoreceptor degeneration [194]. KIAA0586 is required for ciliogenesis. Developing photoreceptors in *kiaa0586* mutant zebrafish have decreased OS development and degenerate rapidly after development [195]. Mutant larvae have significantly reduced ERG responses. TMEM216 is required for cilia assembly and *TMEM216* mutations also cause Joubert syndrome in humans [196,197]. Knockout of *tmem216* resulted in decreased cone OS development, short OSs, disorganized OS discs, and degeneration [198]. *GDF6* encodes a morphogen and its mutation causes LCA and juvenile RP in humans [199]. *gdf6a* mutant zebrafish larvae are functionally blind at 7 dpf as determined by OMR, and have short, dysmorphic cone OSs and ISs, as well as overgrown, disorganized rod OSs and short ISs [199,200]. Photoreceptors do not undergo degeneration in these animals [199].

**Table 5:** Zebrafish mutant models of Leber congenital amaurosis-like photoreceptor disease.

Gene	Photoreceptor Features	Reference
*cluap1*	No OS development, rapid photoreceptor degeneration.	[185]
*gdf6a*	Short, dysmorphic cones and expanded, disorganized rods at 7 dpf.	[199,200]
*ift57*	Short OSs with normal disc stacking at 4 dpf. Central retina photoreceptor degeneration at 5 dpf.	[184,186]
*ift88*	No OS development, rapid photoreceptor degeneration.	[181,182,183,184]
*ift122*	Normal photoreceptor OS development. Degeneration starting at 7 dpf, severe degeneration by 10 dpf.	[187]
*ift172*	No OS development, rapid photoreceptor degeneration.	[181,182,184]
*kiaa0586*	Fewer OSs observed at 3 dpf, photoreceptor degeneration observed at 4 dpf. Decreased photoreceptor function detected at 6 dpf.	[195]
*kif3a*	No OS development and rapid rod photoreceptor degeneration by 5 dpf, subsequent cone degeneration. Extinguished photoreceptor response at 7 dpf.	[161,188,189]
*napbb*	Immediate, severe photoreceptor degeneration; cell death observed at 3 dpf, no distinct photoreceptor layer at 6 dpf, few photoreceptors in ciliary margin.	[191,192]
*tmem216*	Short cilia and disorganized OS discs by 7 dpf, rapid degeneration. Few photoreceptors remaining by 14 dpf.	[198]

#### 4.6.3. Choroideremia

Zebrafish *rep1* knockout larvae have RPE that lacks uniformity with hypertrophic and de-pigmented regions [24] (Table 6). Additionally, the RPE has large vacuoles and accumulation of undigested OS fragments. The photoreceptor layer is disorganized with dysmorphic, functionally defective photoreceptors. The photoreceptor abnormalities appear to result from RPE dysfunction in these animals, as wild-type photoreceptors transplanted into mutant retina still become dysmorphic and degenerate, while *rep1*-negative mutant photoreceptors transplanted into wild-type retinas are morphologically normal. *rep1* mutant zebrafish do not survive to adulthood.

#### 4.6.4. Cone-Rod Dystrophy

*CC2D2A*, *AHI1*, and *ARL13B* encode basal body or cilia-related proteins and their mutation causes forms of Joubert syndrome [201,202,203]. *cc2d2a* knockout zebrafish larvae have disorganized photoreceptor OSs and functional defects [204] (see Table 6 for zebrafish CORD mutants). Of interest, cilia assembly occurs normally in mutants, but trafficking of cilia components is impaired. Zebrafish larvae with *ahi1* mutations have abnormal cone OSs but normal visual function, as assessed by OKR [205]. Cone OS morphology recovers by early adulthood, but cone morphology defects and degeneration are seen in five-month-old animals. At this age, rod photoreceptors have mislocalization of rhodopsin. Zebrafish *arl13b* mutant photoreceptors have short OSs [206]. Knockout *arl13b* larvae do not survive past 9 dpf, so transplantation experiments were performed to assess photoreceptor degeneration. The *arl13b* mutant photoreceptors had noticeable degeneration by 30 dpf. LCA5 is also a ciliary protein and mutations in the *LCA5* gene cause *LCA* [207]. However, knockout of zebrafish *lca5* results in a cone-rod dystrophy phenotype rather than LCA [208]. *lca5* mutant larvae have decreased photoreceptor responses. Cone OS abnormalities are obvious at 1 month of age, while rod OS defects are observed at seven months. Mutations in *PCARE*, a regulator of cilium actin, cause RP [209]. Zebrafish larvae with mutations in *pcare1* have abnormal OS morphology, photoreceptor functional defects, and decreased OKR [210]. Adult *pcare1* mutants have dysmorphic photoreceptor OSs and thin photoreceptor layers, indicative of degeneration. *EYS* mutations typically cause RP in human patients, although *EYS* mutations have also been associated with a cone-rod dystrophy [211,212,213]. The function of EYS is unknown, but a recent study found evidence that EYS localizes to the photoreceptor cilium and may be involved in cilium stability [214]. Zebrafish *eys* mutants have CORD, whereby cones degenerate in six-month-old animals and rod degeneration is observed at 8–14 months [215,216,217]. *BBS2* is a basal body-associated protein necessary for proper ciliogenesis, and mutations in *BBS2* lead to RP and a ciliopathy called Bardet-Biedl syndrome [218,219]. Larval *bbs2* mutants have photoreceptor deficits observed by OKR and short, disorganized photoreceptor OSs [220]. In adulthood, photoreceptor degeneration is observed, along with regeneration of rods but not cones.

*KCNJ13* encodes a channel protein found in the RPE and mutations in *KCNJ13* lead to LCA [221]. Adult zebrafish with mutated *kcnj13* have photoreceptor loss and RPE abnormalities [222]. Specifically, the RPE has increased phagosomes, enlarged mitochondria, and changes in melanosome localization, while cone photoreceptors have abnormal mitochondria and degenerate in aged animals. POMGNT1 is an enzyme that posttranslationally modifies other proteins, and *POMGNT1* mutations cause RP [223]. Zebrafish with *pomgnt1* have cone and rod degeneration at six months of age [224].

#### 4.6.5. Enhanced S-cone Syndrome

A similar phenotype to human patients with enhanced S-cone syndrome is observed in *nr2e3* null zebrafish. Animals lack rod photoreceptors and undergo progressive cone degeneration [225] (Table 6). Interestingly, green and red cones degenerate while UV and blue cones persist. *nrl* mutant larvae also do not develop rods, but intriguingly have increased UV cone photoreceptors [226]. Adult *nrl* mutants unexpectedly have rods, but the rods have abnormal synapses. Whether these rods are functional is unknown.

**Table 6:** Zebrafish mutants of choroideremia, cone-rod dystrophy, and enhanced S-cone syndrome.

Photoreceptor Disease	Gene	Photoreceptor + RPE Features	Reference
Choroideremia	*rep1*	RPE irregularity and pigment loss. Disorganized photoreceptor layers, dysmorphic photoreceptors, and significantly reduced photoreceptor responses at 5 dpf.	[24]
Cone-rod dystrophy	*ahi1*	Disorganized, short OSs, but normal visual function at 5 dpf. Thin photoreceptor nuclear layer, cone degeneration, and dysmorphic rods at 5 months.	[205]
Cone-rod dystrophy	*arl13b*	Short OSs at 4dpf. Cone degeneration observed at 30 dpf in mosaic animals.	[206]
Cone-rod dystrophy	*bbs2*	Short, disorganized photoreceptor OSs and visual deficits at 5 dpf. Photoreceptor degeneration observed in adulthood.	[220]
Cone-rod dystrophy	*cc2d2a*	Dysmorphic photoreceptor OSs and functional defects at 5 dpf.	[204]
Cone-rod dystrophy	*eys*	Progressive photoreceptor loss; cone degeneration observed at 6 months, rod degeneration observed at 14 months	[215,216,217]
Cone-rod dystrophy	*kcnj13*	Increased phagosomes in the RPE at 3 months, enlarged RPE mitochondria at 6 months, and abnormal melanosome localization under dark adaptation at 12 months. Cone mitochondria abnormalities at 6 months and photoreceptor loss at 12 months.	[222]
Cone-rod dystrophy	*lca5*	Photoreceptor functional defects at 7 dpf. Cone OS defects at 1 month, rod OS defects at 7 months, and progressive photoreceptor loss.	[208]
Cone-rod dystrophy	*pcare1*	Dysmorphic OSs and dysfunctional photoreceptors at 5 dpf. Abnormal OS morphology and thinner photoreceptor layer at 6 months.	[210]
Cone-rod dystrophy	*pomgnt1*	Reduction in cones and rods at 6 months.	[224]
Enhanced S-cone syndrome	*nr2e3*	No rods. Green and red cone degeneration started at 1 month.	[225]
Enhanced S-cone syndrome	*nrl*	Rods fail to develop and UV cones are over-abundant in larvae. Adult zebrafish surprisingly have rods, but with synaptic defects.	[226]

#### 4.6.6. Cone Dystrophy

*PROM1* plays a critical role in outer segment disc morphogenesis and *PROM1* mutations cause a variety of photoreceptor diseases, such as RP, CORD, and macular degeneration [227,228,229]. Zebrafish with *prom1b* mutations have a cone degeneration, starting with red and green cones and affecting blue and UV cones soon after [230] (Table 7; see for list of zebrafish mutant models of cone dystrophy). While rods do not appear to degenerate in *prom1b* mutants, they have overgrown OSs. *CEP290* encodes a centrosomal protein and mutations are the most frequent cause of LCA [17]. Interestingly, *cep290* mutant zebrafish have cone OS disorganization and degeneration in adulthood that is severe by 12 months, but normal rod OS morphology [231]. Why *cep290* results in an adult onset, cone-specific phenotype in zebrafish is unknown, but may be due to alternative photoreceptor centrosomal proteins in zebrafish.

*PDE6C* is an essential component of the cone phototransduction pathway and results in cone dystrophy when mutated [232]. *pde6c*-deficient zebrafish have rapid cone degeneration as well as dysmorphic, degenerate rods in larval stages [49,192]. In adulthood, mutants have severe cone degeneration but normal rod photoreceptors. AIPL1 is required for maintenance of photoreceptor phototransduction machinery and *AIPL1* mutations can lead to LCA, RP, and CORD [233,234]. Zebrafish have two copies of this gene, *aipl1a*, which is expressed in rods and potentially UV cones, and *aipl1b*, which is expressed in all cones [235]. Zebrafish with mutations in *aipl1b* have progressive cone degeneration starting in larval stages, and by early adulthood have only a few single cones remaining [235]. Rod photoreceptors are unaffected.

Mutations in the retinal voltage-gated calcium channel subunit gene, *CACNA2D4*, lead to cone dystrophy [236]. Zebrafish have two paralogs of *CACNA2D4*: *cacna2d4a* and *cacna2d4b* [96]. Knocking out each paralog individually does not impact photoreceptor function in early development, but double knockout animals had reduced cone function [96]. ATP6V0E1 is a subunit of a proton pump and *atp6v0e1* mutant zebrafish larvae have RPE pigment loss, outer retinal holes, visual defects determined by OKR, photoreceptor functional deficiencies assessed by ERG, and short OSs [237]. Rods were not assessed in these animals.

WRB is involved in recruitment of proteins to the endoplasmic reticulum, although its exact role in photoreceptors is unclear [238]. *wrb* mutant zebrafish larvae have holes in their photoreceptor layer, decreased or absent OMR, photoreceptor function deficits, and short cone ribbon synapses in early life [239,240]. Rods have not been investigated in these animals. Mutations in *WRB* have not been associated with human disease.

#### 4.6.7. Achromatopsia

GNAT2 is an essential component of the cone phototransducation pathway and pathogenic variants in *GNAT2* lead to achromatopsia [241]. *gnat2* mutant zebrafish have cone dysfunction but the photoreceptors do not have overt morphological defects [242] (see Table 7 for mutant zebrafish achromatopsia models). Electrophysiological assessment suggested that *gnat2* mutant cones are ~1000 times less sensitive than wild-type cones. The rods are unaffected in these animals.

Pathogenic mutations in *CACNA1F* cause CORD and congenital stationary night blindness, a non-progressive condition characterized by rod dysfunction [243,244,245]. CACNA1F protein is a subunit of a voltage-gated calcium channel required at the photoreceptor synapse [246]. Zebrafish *cacna1fa* is cone-specific and mutant retinas have thin photoreceptor synaptic layers and decreased photoreceptor responses [97]. *cacna1fa* mutant cones do not develop synaptic ribbons. Similarly, zebrafish *synj1* mutants have a thin photoreceptor synaptic layer and abnormal cone pedicles with unanchored synaptic ribbons [247,248,249]. The cone IS and OS appear normal [247,248,249]. Mutant zebrafish do not survive long enough to develop appreciable numbers of functional rods, but a transplantation study demonstrated that *synj1* is required for cone synapse formation but not rod synapses [249]. A related phenotype is seen in *per2* knockout zebrafish; *per2* mutant larvae have an abnormal VMR, untethered ribbon synapses, and decreases in cone opsin expression which may indicate cone degeneration [250]. Animals have not been aged for further investigation of cone and rod viability.

#### 4.6.8. Age-Related Macular Degeneration

AMD models are challenging to generate as AMD is a multifactorial condition. However, there are zebrafish models with features of AMD. Recently, *rp1l1* mutant zebrafish were reported to have progressive photoreceptor dysfunction and subretinal drusenoid deposits [170] (Table 4). This is the first report of subretinal drusenoid deposits in zebrafish, and may provide insight into how drusenoid deposits develop and impact photoreceptor function. In addition, models of neovascularization are relevant to wet AMD. Zebrafish larvae with mutations in *vhl* present with retinal neovascularization, leaky vessels, and develop oedema and retinal detachment [251] (Table 7).

**Table 7:** Zebrafish mutant models of cone dystrophy, achromatopsia, and retinal neovascularization.

Photoreceptor Disease	Gene	Photoreceptor, RPE, or Vascular Features	Reference
Achromatopsia	*cacna1fa*	Thin photoreceptor synaptic layer. Photoreceptor functional defects at 5–6 dpf. Cone synapses do not develop synaptic ribbons.	[97]
Achromatopsia	*gnat2*	Cone functional defects under dim to moderate light intensities.	[242]
Achromatopsia	*per2*	Visually-mediated behaviour deficiencies at 5–6 dpf. Unanchored cone ribbon synapses and decreased cone opsin expression.	[250]
Achromatopsia	*synj1*	Thin photoreceptor synaptic layer and abnormal, unanchored cone ribbon synapses at 6 dpf.	[247,248,249]
Cone dystrophy	*aipl1*	Thin photoreceptor layer and OS defects at 7 dpf. Severe cone degeneration by 3 months.	[235]
Cone dystrophy	*atp6v0e1*	Hypopigmented RPE, reduced or absent visual response, decreased photoreceptor function, short OSs, and outer retinal holes at 5 dpf.	[237]
Cone dystrophy	*cacna2d4a + cacna2d4b*	Mild cone dysfunction at 20 dpf.	[96]
Cone dystrophy	*cep290*	Cone OS abnormalities and degeneration at 3 months, severe degeneration at 12 months. Normal rods.	[231]
Cone dystrophy	*pde6c*	Cone degeneration at 4 dpf and rod subsequently become dysmorphic and degenerate in the larval retina. At 3 months, cones are degenerate and largely missing, rods are normal.	[49,192]
Cone dystrophy	*prom1b*	Progressive cone degeneration. Reduced red and green cones at 7 dpf; reduced UV and blue cones at 1 month. Overgrown rod OSs.	[230]
Cone dystrophy	*wrb*	Holes in the photoreceptor layer at 4 dpf. Vision defects at 5 dpf, photoreceptor function defects at 4–5 dpf. Small cone ribbon synapses	[239,240]
Retinal neovascularization	*vhl*	Retinal neovascularization, leaky blood vessels, retinal oedema, and retinal detachment at 7–8 dpf.	[251]

### 4.7. Relevance to Human Disease

In order to understand and prevent photoreceptor disease, a keen knowledge of how photoreceptor development, function, and maintenance is required. Zebrafish have provided additional information about factors involved in these aspects, as lines with mutations in genes not associated with human photoreceptor disease, potentially due to lethality, have impaired or degenerate photoreceptors. Additionally, the cellular abnormalities and cause of photoreceptor death can be investigated in these models. For example, genetically mosaic zebrafish generated by transplantation experiments with zebrafish *rep1* mutants determined that *rep1* loss in photoreceptors does not lead to photoreceptor degeneration, but rather that *rep1* is required for normal RPE function, and photoreceptors die due to RPE dysfunction in *rep1* mutant retina [24].

Models of cone disease are desperately needed, as preservation of the high-acuity central vision is a priority for patients and physicians. Nocturnal rodent models are not an ideal system for these conditions, as they have a low density of cones and their cones have some properties that are distinct from human cones, such as expression of multiple opsins in a single cell. Indeed, some mouse models of cone diseases do not have the expected cone degeneration phenotype [252]. The zebrafish retina allows for the investigation of cone diseases, and the numerous genetic models provide the opportunity to determine disease mechanisms based on the mutated gene.

In addition, the cone-rich zebrafish retina enables the assessment of how rod loss impacts cone photoreceptors. Cone persistence post rod death in adult zebrafish RP models permits determination of how cones respond to rod loss and the factors that influence their health when neighbouring photoreceptors degenerate. Additionally, while cones appear viable post rod loss in RP zebrafish models, *pde6c* mutant larvae have rod degeneration post cone loss despite the cone-specific genetic lesion [49,192]. This suggests that the minority photoreceptor type is inherently susceptible to death, as the cone-dominant larval zebrafish retina undergoes rod degeneration post cone loss while the rod-dominant human retina experiences cone degeneration post rod loss. The *pde6c* mutants can therefore be utilized to determine why the minority photoreceptor type is sensitive to degeneration and provide avenues to preserve the cells, which is relevant to efforts aimed at protecting cones in late-stage RP.

Interestingly, several zebrafish models of photoreceptor disease have red and green cone degeneration prior to UV and blue cone degeneration [166,180,225,230,235]. Whether this is due to structural distinctions between double and single cones, or whether there are specific features of longer-wavelength sensitive cones that make them more susceptible to cell death than short wavelength cones, is unknown. Further assessment is required and may inform macular degeneration pathologies. The central macula is comprised of primarily red and green cones, and understanding what makes these cones vulnerable to disease is important for preventing AMD.

Drusen and subretinal drusenoid deposits are characteristic features of AMD but how they develop and their effect on photoreceptor health is unknown. Animal models of drusen and drusenoid deposits are difficult to develop and there a few photoreceptor disease models that acquire them. Subretinal drusenoid deposits in particular are poorly investigated, as they are relatively newly characterized. A zebrafish model of photoreceptor disease with accompanying subretinal drusenoid deposits provides the opportunity to investigate how these lipid accumulations develop in vivo [170].

## 5. Contribution to Therapeutics

Zebrafish provide a model to test therapeutic strategies and pharmaceuticals. Numerous disease models combined with the small size of zebrafish larvae and visual tools, such as VMR, OMR, and OKR, allow for screening of compounds. Below, the contributions from zebrafish models are outlined.

### 5.1. Pharmaceuticals

#### 5.1.1. Anti-Angiogenic Compounds

Zebrafish models of retinal neovascularization, such as the hypoxia-induced model and *vhl* mutants, provide the opportunity to screen for anti-angiogenic compounds that could be employed in patients with wet AMD. Indeed, Van Rooijen and colleagues found that treating *vhl* mutants with vascular endothelial growth factor receptor (VEGFR) tyrosine kinase inhibitors typically used in cancer treatment successfully blocked pathogenic angiogenesis [251], which could be further tested in mammalian models of retinal neovascularization.

#### 5.1.2. Histone Deacetylase Inhibition

Cellular stress markers can activate cell death pathways, resulting in apoptosis, or trigger elimination by immune cells. This can result in loss of cells that were stressed, but otherwise functional, and has been observed in the retina [253]. Overactivation of histone deacetylases (HDACs) has been detected in models of photoreceptor degeneration [254]. Indeed, HDAC inhibitors have been effective for treating neuronal pathologies in vitro and in animal models of CNS disease, including photoreceptor degeneration models [255,256,257]. However, it is important to note that there are also HDACs that inhibit photoreceptor death after light injury [258]; it is therefore important to target specific HDACs.

HDAC6 inhibition has been reported to prevent neurodegeneration in peripheral neuropathy models [259]. In the *atp6v0e* mutant zebrafish model of cone photoreceptor disease, HDAC6 inhibitors successfully restored OKR, decreased the number of apoptotic cells in the photoreceptor layer, and increased OS persistence [237,260,261]. Similar results are observed in a mouse model of photoreceptor disease [260,261], further supporting HDAC6 as a potential therapeutic target for treating photoreceptor degenerations.

#### 5.1.3. Novel Compound Screening

Zebrafish models of photoreceptor disease provide a platform for discovery of novel neuroprotective drugs. Zebrafish have been employed for numerous large drug screens, such as screens to identify compounds that impact neuronal activity [262,263]. Zebrafish can therefore be utilized to identify novel compounds in large-scale screens. Through behavioural screens and histological approaches, several compounds have been identified as protective against photoreceptor dysfunction and degeneration in zebrafish.

Ganzen and colleagues utilized Tg(*rho:hsaRHO-Q344X*) and transgenics with NTR-ablatable rods to screen a library of potential drugs for potential RP treatment [264] (bioRxiv preprint). VMR assessment identified carvedilol as beneficial, and carvedilol-treated Tg(*rho:hsaRHO-Q344X*) larvae had increased rod survival compared to control animals. As RP is the most common inherited photoreceptor disease, a pharmacological treatment may have applicability to many patients experiencing progression vision loss.

Cone degeneration in *pde6c* mutant zebrafish larvae occurs through nectroptosis, while the subsequent rod degeneration is apoptosis-mediated [265]. Blocking necroptosis factors using necrostatin-1s or necrosulfonamide at 10 hpf until 7 dpf results in a reduction in cone death [265]. However, whether vision is preserved due to necrostatin-1s or necrosulfonamide treatment is unknown. Further investigation is required, but pharmacological repression of necroptosis has applicability to many different photoreceptor diseases that involve necroptosis pathways.

Studies using zebrafish models of photoreceptor disease have also identified schisandrin B and gypenoside, plant-derived compounds with antioxidant and anti-inflammatory properties, as potential neuroprotective compounds [266,267,268,269,270,271]. These examples highlight the benefits of utilizing zebrafish models of photoreceptor disease to screen novel therapeutics and specific drugs.

#### 5.1.4. Oculotoxicity

Many drugs have retinal toxicity, meaning that prolonged exposure can result in retinal damage and vision loss. Zebrafish have provided a relatively high-throughput tool to assess retinal toxicity of pharmaceuticals. Deeti and colleagues used zebrafish larvae to develop a methodology for assessing the impact of pharmaceuticals on the zebrafish visual system [272]. Thereby, 3 dpf, zebrafish larvae were treated with compounds for 2 days in 48-well plates, and then assessed for visual impairment using VMR and OKR. 5/6 known oculotoxic drugs reduced the visual responses of zebrafish. However, all of the oculotoxic drugs resulted in a reduction in zebrafish touch response, suggesting defects in other sensory neurons. Therefore, zebrafish provide a viable tool for screening drugs for retinal toxicity.

### 5.2. Antisense Oligonucleotide Therapy

Antisense oligonucleotide therapy can block aberrant splicing in genes with mutations in splice donor or acceptor sites. A frequent Usher syndrome-causing *USH2A* mutation results in incorporation of a psuedoexon and truncation of the USH2A protein [273]. Morpholino treatment successfully prevented aberrant splicing in mosaic zebrafish with *ush2a* containing a CRISPR/Cas-introduced region of human mutant *USH2A* [274]. In addition, this group recently investigated morpholino-induced skipping of a mutated exon in *ush2a* mutant zebrafish [275] (preprint); specifically, they skipped mutant exon 13, which is commonly mutated in Usher syndrome [178,276]. Exon skipping was successful and partially restored protein expression [275] (preprint). This therapeutic approach of using antisense oligonucleotides to partially restore *USH2A* function by skipping the problematic exon 13 is currently in clinical trial (ID: NCT03780257).

### 5.3. Photoreceptor Regenerative Pathways

Zebrafish can regenerate lost retinal neurons, including photoreceptors [277]. Investigating the zebrafish regenerative response provides information on activation of endogenous retinal stem cells as well as factors that promote photoreceptor differentiation and integration of newly generated cells, which is invaluable for effective development and deployment of stem cell therapies.

Zebrafish have two distinct stem cell pools: the ciliary marginal zone (CMZ) and Müller glia.

#### 5.3.1. Ciliary Marginal Zone

The CMZ is a pool of retinal stem cells located at the peripheral edges of the retina. In many species, this pool becomes inactive once the retina has matured. However, in many vertebrates, such as zebrafish and other species that undergo indeterminate growth, the CMZ self-renews and constantly adds new retinal neurons at the edges of the retina. The CMZ contains both retinal stem cells, which can divide indeterminately, and retinal progenitor cells, which can only divide a certain number of times and lose contact with the CMZ as differentiation occurs [104,277,278,279]. Retinal stem cells sit in the edges of the CMZ, and typically undergo asymmetrical division to produce a retinal stem cell and a retina progenitor cell [104,277,278,279].

Mammals have a CMZ-like region that gives rise to some retinal neurons during development [280], although whether it has the capacity to produce photoreceptors is unclear. In the mature retina, these retinal margin cells seem to lose neurogenesis capacity or become quiescent. However, there are cells with stem cell-like properties in the mammalian/human peripheral retinal margin net to the pars plana; specifically, Müller glia and ciliary epithelium cells that express neural progenitor markers [281,282,283]. Whether these cell populations could be stimulated to enter a stem cell-like fate in the damaged retina requires further investigation.

#### 5.3.2. Müller Glia

Müller glia are support cells that play many essential roles in the retina, including ion regulation, neurotransmitter uptake and degradation, cellular debris removal, and neuronal insulation. In zebrafish and other teleost fishes, Müller glia also act as endogenous stem cells by undergoing de-differentiation, proliferation, and specification to produce many different retinal neuron types, including photoreceptors [281,282,283]. Zebrafish Müller glia division is asymmetrical, allowing for replenishment of lost retinal neurons while maintaining the stem cell pool [99,104,107,279,284,285,286,287]. As mentioned above, there is a threshold of damage that must be met to trigger the Müller glial regenerative response, which has been observed in NTR ablation studies [60,125,126,127,128,129]. Interestingly, some mutant zebrafish have cone degeneration but Müller glia do not undergo proliferation to regenerate the lost photoreceptors [220,231]. Further investigation into one of these lines, the *bbs2* mutants, found that the endogenous Müller glia do have the capacity to regenerate retinal neurons in *bbs2* mutant retina, as evident by light ablation experiments [220]. Why photoreceptor degeneration triggers a regenerative response in some mutant models but not others is unclear, but may be related to the speed of degeneration or an inflammatory requirement for Müller glia activation.

Zebrafish Müller glial behaviour must be carefully controlled in order to prevent aberrant proliferation and production of unneeded neurons. In the uninjured retina, the reprograming pathways of Müller glia are inhibited [288]. After retinal injury, Müller glia respond to factors released by damaged cells—including TNFα [289]—and undergo reprogramming, followed by proliferation and neurogenesis. Studying the regenerative pathways in zebrafish has provided insight into stem cell and fate-determining factors that can be utilized to generate photoreceptor precursor cells for transplantation.

As Müller glia are interspersed within the human retina, how the stem cell behaviour of Müller glia is activated and whether mature mammalian Müller glia can be re-programmed to a stem cell fate to treat retinal degenerations is an area of active interest. Human Müller glia respond to damage and have stem cell-like characteristics, but do not naturally undergo neurogenesis in vivo [281,290]. Müller glia proliferation is rare in the mammalian retina, usually associated with scar formation [291]. In culture, human Müller glia treated with growth and differentiation factors can take on stem cell characteristics and differentiate into rod photoreceptors [281,290,292]. Rod photoreceptors derived from human Müller glia cultures have successfully been introduced into a rat model of RP. These cells are able to migrate into the ONL and restore some visual function [293]. Further investigations into Müller glial response to photoreceptor loss, photoreceptor cell differentiation, and integration will be essential for application of stem cell therapies in human disease, and zebrafish provide this unique avenue. For in-depth reviews of photoreceptor regeneration in zebrafish and how these mechanisms relate to mammalian systems, please see [145,294,295,296,297,298].

## 6. Conclusions

Zebrafish provide the unique opportunity to model photoreceptor disease in a cone-rich retina, similar to the human macula. There are inducible, transgenic, and mutant zebrafish models that encompass the entire spectrum of photoreceptor disease. These models can be further utilized to elucidate disease mechanism, test therapeutics, and screen novel compounds. In addition, the robust regenerative capacity of the zebrafish retina allows for the characterization of endogenous stem cells.

## Data Availability

Data sharing not applicable.
